# Impact of High-Molecular-Weight Hyaluronic Acid on Gene Expression in Rabbit Achilles Tenocytes In Vitro

**DOI:** 10.3390/ijms23147926

**Published:** 2022-07-18

**Authors:** Iris Miescher, Petra Wolint, Christine Opelz, Jess G. Snedeker, Pietro Giovanoli, Maurizio Calcagni, Johanna Buschmann

**Affiliations:** 1 Division of Plastic Surgery and Hand Surgery, University Hospital Zurich, Sternwartstrasse 14, 8091 Zurich, Switzerland; iris.miescher@usz.ch (I.M.); petra.wolint@usz.ch (P.W.); christine.opelz@web.de (C.O.); pietro.giovanoli@usz.ch (P.G.); maurizio.calcagni@usz.ch (M.C.); 2 Orthopaedic Biomechanics, University Clinic Balgrist, Forchstrasse 340, 8008 Zurich, Switzerland; jess.snedeker@hest.ethz.ch

**Keywords:** rabbit tenocytes, tenascin C, tenomodulin, Mohawk, IL-6, TNF-α, PAR-2, LPS, LipoxinA4, fibronectin

## Abstract

(1) Background: Surgical tendon repair often leads to adhesion formation, leading to joint stiffness and a reduced range of motion. Tubular implants set around sutured tendons might help to reduce peritendinous adhesions. The lubricant hyaluronic acid (HA) is a viable option for optimizing such tubes with the goal of further enhancing the anti-adhesive effect. As the implant degrades over time and diffusion is presumed, the impact of HA on tendon cells is important to know. (2) Methods: A culture medium of rabbit Achilles tenocytes was supplemented with high-molecular-weight (HMW) HA and the growth curves of the cells were assessed. Additionally, after 3, 7 and 14 days, the gene expression of several markers was analyzed for matrix assembly, tendon differentiation, fibrosis, proliferation, matrix remodeling, pro-inflammation and resolution. (3) Results: The addition of HA decreased matrix marker genes, downregulated the fibrosis marker α-SMA for a short time and slightly increased the matrix-remodeling gene MMP-2. Of the pro-inflammatory marker genes, only IL-6 was significantly upregulated. IL-6 has to be kept in check, although IL-6 is also needed for a proper initial inflammation and efficient resolution. (4) Conclusions: The observed effects in vitro support the intended anti-adhesion effect and therefore, the use of HMW HA is promising as a biodegradable implant for tendon repair.

## 1. Introduction

Surgical tendon rupture repair suffers from two main problems [1]. On the one hand, the healing tendon tissue does not usually regain its full strength from before the injury, and scar formation often leads to inferior mechanical properties [2]. On the other hand, adhesion formation with the surrounding tissue hampers proper motion and may lead to joint stiffness [3]. The problem of adhesion formation is particularly pronounced in intrasynovial tendons [4], such as the flexor tendons of the hand; however, it has also been reported for the Achilles tendon (AT) [5,6]. When the tendon and the tendon sheath are injured, a mismatch in the fibrinolytic balance during wound healing may result in the formation of excessive fibrin that, in turn, acts like a glue and leads to unwanted adhesions [7].

The gliding of tendons in their sheath is enabled by a specific composition of the synovial fluid [8,9]. Lubricating biomolecules, such as lubricin [10], glycosaminoglycans and hyaluronic acid (HA) [11], are prerequisite components for proper gliding with low friction. HA is a key player among these biolubricants [12]. Chemically, HA is a polysaccharide, composed of the disaccharide monomer glucuronic acid and N-actetylglucosamine. At a neutral pH, HA is negatively charged, which explains its high hydrophilicity and electrical conductivity. The distinct rheological characteristics of aqueous HA formulations include high viscosity and high surface tension [13].

Besides acting as an excellent lubricant in the synovial fluid, HA is a major component of the extracellular matrix (ECM) [14] and a regulatory key player during wound healing [15]. The biological effects of HA depend on its sugar chain length [16]. High-molecular-weight (HMW) HA (>10^6^ Da) has been reported to act in anti-angiogenic and anti-inflammatory ways [17], while low-molecular-weight (LMW) HA (2 × 10^4^–10^6^ Da) is rather pro-angiogenic and pro-inflammatory [12]. The formation and degradation of HA sugar chains are in a delicate balance, and are caused by enzymes hyaluronan synthases and hyaluronidases, respectively. In addition, radical oxygen species cause HA fragmentation and lead to the further degradation of HA in the lymph nodes [12].

An anti-adhesion implant in the form of an electrospun polymer tube [18] placed around a fully transsected and sutured rabbit AT has been shown to significantly reduce the adhesion extent by 20% [19]. However, to improve tendon healing, it is necessary to reduce adhesion further, which is why the aim is to use HA as a biolubricant to enhance the extent of anti-adhesion effectiveness. The idea is to incorporate HA via electrospinning in one layer of the implant [20] so that it would act as a boundary lubricant and simultaneously as a fluid lubricant when applied in our full transection rabbit AT model [21]. However, before such further optimization of the implant material can be performed, it is of central importance to determine the effects of HA on the rabbit tenocytes in vitro.

For this study, a HMW HA with a wide range of sugar chain lengths was chosen (1.01–1.8 × 10^6^ Da) and the medium for the rabbit AT tenocyte cultures was supplemented with a physiological HA concentration of 1.4 mg/mL [22]. The proliferation was quantified for three rabbit donors and compared to a normal culture medium (control). Moreover, the dynamics of gene expression were assessed. After 3, 7 and 14 days of exposure, the tendon, remodelling, inflammatory and resolution markers were measured by quantitative real-time PCR. Finally, in order to elucidate the impact of HA on tenocyte ECM formation, the protein expression of collagen 1 and fibronectin was determined by immunocytochemistry.

The aim of this in vitro study was to determine the effects of HMW HA supplementation on rabbit Achilles tenocytes with respect to (i) proliferation, (ii) the gene expression profile, (iii) ECM formation and (iv) inflammatory response.

## 2. Results

### 2.1. Proliferation of Rabbit Achilles Tenocytes

**Figure 1:**
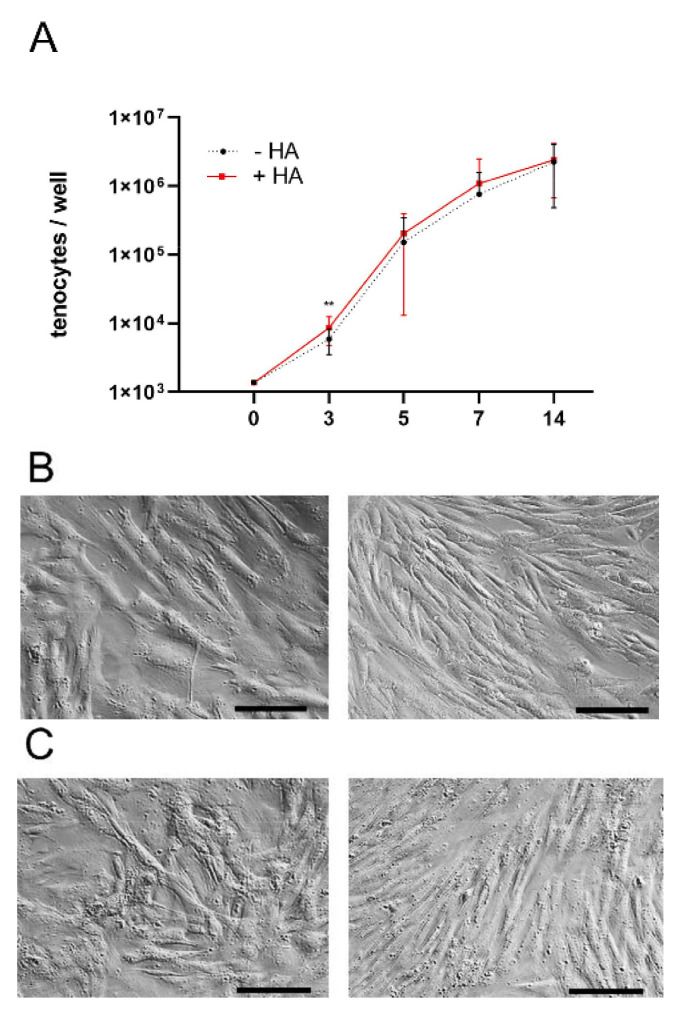
Proliferation of rabbit Achilles tenocytes in vitro (n = 3 rabbits) with or without HA (**A**). Values denote means and error bars stand for standard deviations. Tenocytes after 2 weeks of culture without (**B**) or with HA supplementation (**C**). Scale bar: 100 µm. *p* < 0.01 (**).

As a first step towards determining the effects of HMW HA (1.1–1.8 MDa) on rabbit Achilles tenocytes, an alamarBlue™ cell viability assay was performed and the proliferation of the cells was assessed with or without the addition of 1.4 mg/mL HA to the culture medium. As can be seen in Figure 1, the HMW HA had negligible effects on the proliferation of the cells, as the growth curves obtained for the cells extracted from the three rabbits were very similar. The decay of long chain HA into smaller fragments was taken into consideration by changing the culture medium every day. The doubling times were 13 ± 4.5 h and 14 ± 4.5 h for the tenocytes with HA and without HA, respectively, based on a population increase between day 3 and day 5 and calculated according to a standardized growth curve. Except on day 3, no significant differences were observed for these two groups.

### 2.2. Gene Expression of Extracellular Matrix Markers

**Figure 2:**
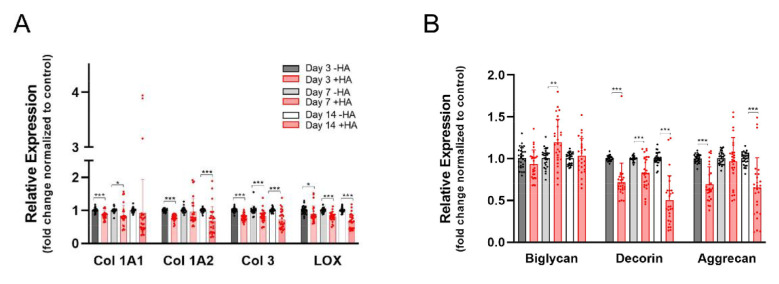
Extracellular matrix components *collagen IA1 (Col IA1)*, *collagen IA2 (Col 1A2)* and *collagen 3 (Col 3)* as well as *lysyl oxidase (LOX)* responsible for crosslinking collagen fibers (**A**). Proteoglycans occurring in tendon tissue; *biglycan*, *decorin* and *aggrecan* (**B**). Stars indicate pairwise comparison probability in Student’s *t* test, where HA was compared to the control: *p* < 0.05 (*), *p* < 0.01 (**) and *p* < 0.001 (***).

The gene expression of collagens revealed a significant downregulation of *Col IA1*, *1A2* and *3* over the time course of 14 days (~0.7-fold) in HA-treated cells compared with untreated cells. (Figure 2A). The *LOX* expression paralleled those findings. The proteoglycans *decorin* and *aggrecan* were also reduced to a level of 0.6-fold expression during 14 days, while *biglycan* experienced and upregulation on day 7 (1.2-fold) and stayed otherwise constant (Figure 2B).

### 2.3. Gene Expression of Tendon Markers and Pro-Fibrotic Alpha-SMA

**Figure 3:**
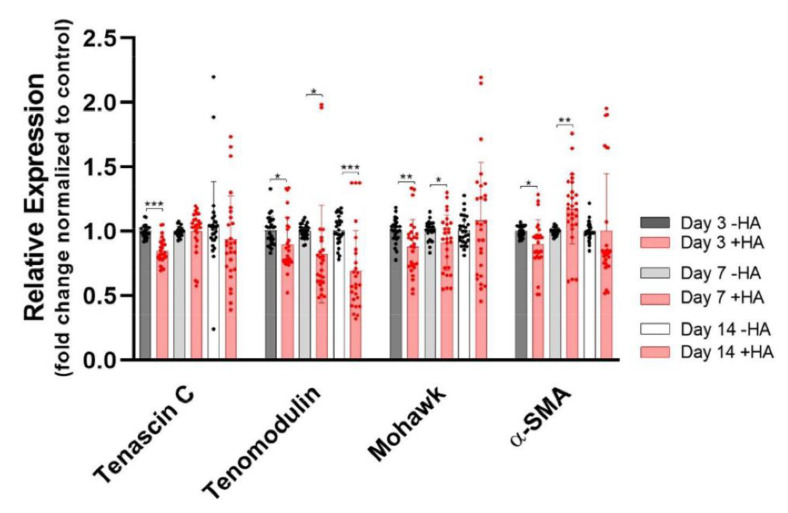
Tendon markers and fibrotic marker *α-SMA*. Stars indicate pairwise comparison probability in Student’s *t* test, where HA was compared to the control: *p* < 0.05 (*), *p* < 0.01 (**) and *p* < 0.001 (***).

The typical tendon genes *tenascin C* (*TNC*), *tenomodulin* (*TNMD*) and *Mohawk* (*MKX*) experienced a slight downregulation on day 3 compared to the control (Figure 3). While *TNMD* experienced further downregulation on days 7 and 14 (final downregulation by a factor of ~0.7), *TNC* and *MKX* showed only minor differences. As for the pro-fibrotic marker *alpha smooth muscle actin (α-SMA*), it also experienced a slight downregulation on day 3; however, on day 7, it reversed to a slight upregulation and ended up at a similar expression level on day 14. Overall, the changes were small in all of the markers (in the range of 0.7- to 1.2-fold).

### 2.4. Remodeling Markers and Proliferation Marker ki67

**Figure 4:**
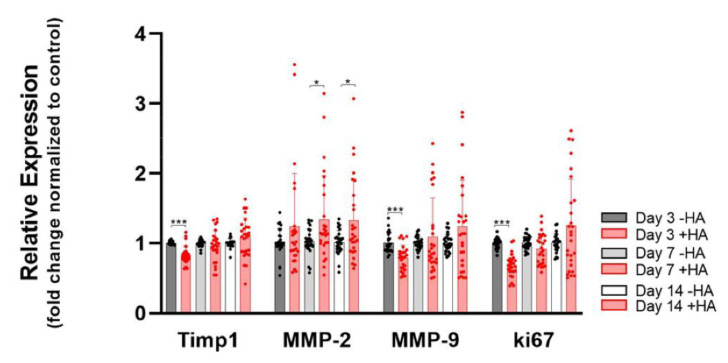
Remodeling markers and proliferation marker *ki67*. Stars indicate pairwise comparison probability in Student’s *t* test, where HA was compared to the control: *p* < 0.05 (*) and *p* < 0.001 (***).

The lubricant HA might also have an impact on the remodeling of tendon tissue. Therefore, we assessed the gene expression of two matrix metalloproteases, *MMP-2* and *MMP-9*, respectively, as well as one tissue inhibitor of matrix metalloprotease, *Timp1*. Figure 4 shows a small but significant increase in the *MMP-2* expression on days 7 and 14, while *MMP-9* experienced a slight downregulation on day 3, but then stayed constant until day 14 with no further significant differences when compared with the control. The dynamics of *Timp1* gene expression in the presence of HA were very similar to those of *MMP-9*, with a significant small downregulation on day 3 and no further significant changes up to 14 days.

While in the alamarBlue™ assay, HA did not show any impact on the proliferation except on day 3 (Figure 1), the gene expression of the proliferation marker *ki67* was downregulated on day 3 (0.7-fold). On days 7 and 14, however, *ki67* was not significantly affected anymore (Figure 4).

### 2.5. Pro-Inflammatory and Resolution Markers

**Figure 5:**
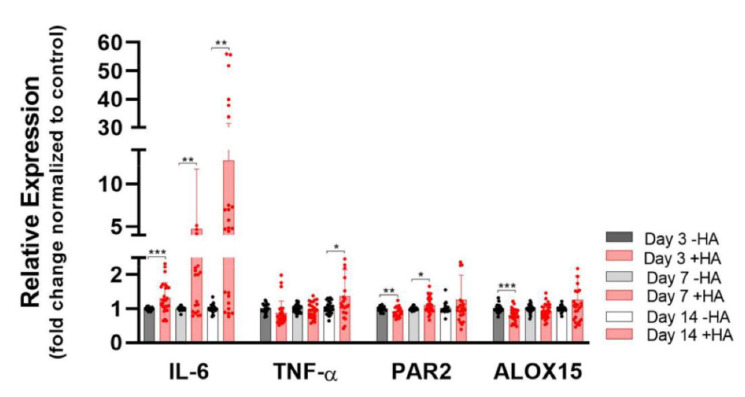
*IL-6*, pro-inflammatory markers (*TNF-α* and *PAR2*) and resolution marker (*ALOX 15*) in the presence of HA compared with the control (without HA). Stars indicate pairwise comparison probability in Student’s *t* test, where HA was compared to the control: *p* < 0.05 (*), *p* < 0.01 (**) and *p* < 0.001 (***).

As can be seen in Figure 5, *IL-6* was significantly increased over time in the presence of HA and none of the two pro-inflammatory markers were affected to such an extent as *IL-6*. While *TNF-α* showed a small upregulation only on day 14, for *PAR-2*, we observed a tiny downregulation on day 3, followed by a slight upregulation on day 7, without further changes compared to the non-HA control on day 14.

The pro-resolving marker *ALOX15* experienced a small but highly significant downregulation on day 3. After that time point, no significant changes were observed until day 14.

**Figure 6:**
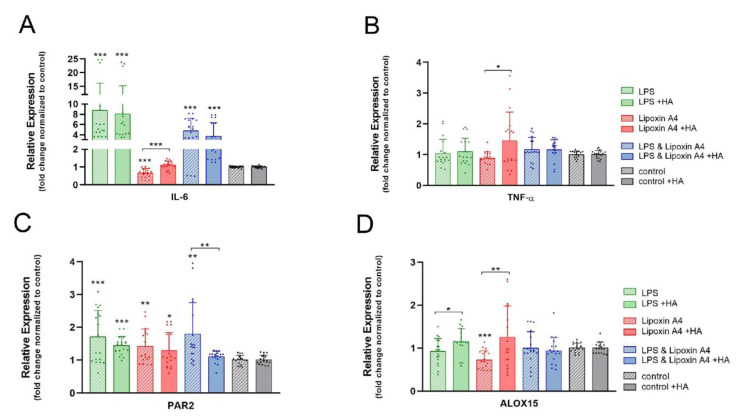
*IL-6* (**A**), pro-inflammatory markers *TNF-α* (**B**) and *PAR2* (**C**) and pro-resolution marker *ALOX15* (**D**) stimulated with LPS, Lipoxin A4 or both molecules compared to the control without stimulation. Stars indicate pairwise comparison probability in Student’s *t* test comparing tenocytes with or without inflammatory and pro-resolving stimulation. Stars above a line indicate pairwise comparison probability in Student’s *t* test comparing tenocytes with the same stimulation, but in a different culture medium (without or with HA): *p* < 0.05 (*), *p* < 0.01 (**) and *p* < 0.001 (***).

In order to mimic an inflammatory milieu, a short-time experiment with lipopolysaccharide (LPS) stimulation of tenocytes was conducted, while mimicry of a pro-resolution milieu was achieved by the addition of Lipoxin A4 to the culture medium. Finally, an initial pro-inflammatory environment with LPS for 4 h, followed by a mimicked resolution by Lipoxin A4 stimulation, was created in a third combination experiment. These experiments were either performed without HA or in the presence of HA, and the gene expression of the markers involved in immune response was analyzed (Figure 6).

The gene expression level of *IL-6* was significantly increased by LPS stimulation (nine-fold without and eight-fold with HA supplementation, respectively). The expression levels were lower when Lipoxin A4 was added after the LPS treatment (5-fold or 3.5-fold with HA). The addition of Lipoxin A4 led to the downregulation of *IL-6* (0.8-fold) in the control group and remained unaffected in the HA-treated cells. (Figure 6A).

Only the combination of LPS and Lipoxin A4 showed a slight but significant upregulation of *TNF-α* under HA supplementation (1.5-fold), while the other conditions did not influence *TNF-α* expression (Figure 6B).

The *PAR2* levels in the control medium were upregulated independently from the stimulation. The tenocytes with HA supplementation showed weaker *PAR2* upregulation than the cells in the control medium for all stimulations. Moreover, after LPS and Lipoxin A4 stimulation, the *PAR2* levels did not differ from those of the unstimulated tenocytes (Figure 6C).

The exposition of rabbit tenocytes to Lipoxin A4 without HA supplementation led to the significant downregulation of the pro-resolution marker *ALOX15*, while other stimulations did not influence the cells in the control medium. Stimulation with LPS or Lipoxin A4 led to higher gene expression levels for *ALOX15* in the tenocytes with HA supplementation compared to those in the cells cultured in HA medium without stimulation. These effects were not significant, but comparison of the tenocytes with LPS or Lipoxin A4 stimulation showed significant higher *ALOX15* levels in the HA-supplemented medium compared to the stimulated tenocytes in the control medium (Figure 6D).

### 2.6. Immunocytochemistry for Collagen I and Fibronectin

**Figure 7:**
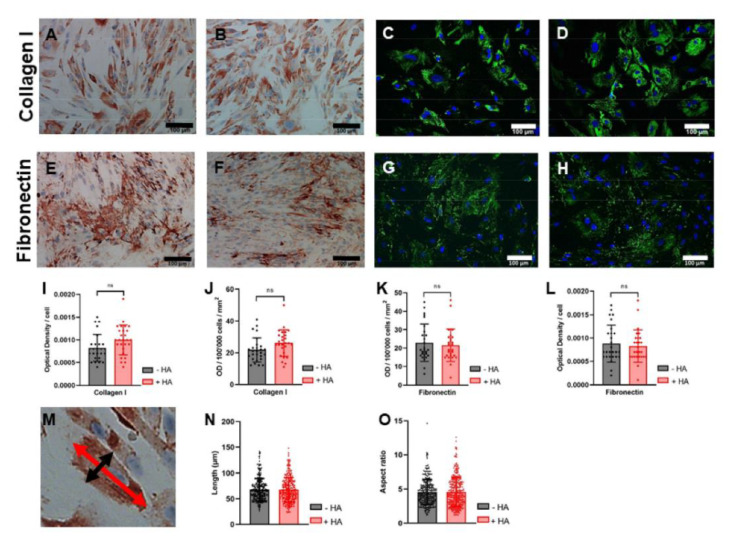
DAB-stained rabbit tenocytes for collagen I (**A**,**B**) and fibronectin (**E**,**F**), and fluorescence staining for collagen I (**C**,**D**) and fibronectin (**G**,**H**), without HA supplementation (**A**,**C**,**E**,**G**) or with HA supplementation (**B**,**D**,**F**,**H**). Quantitative results of optical densities of collagen I (**I**,**J**) and fibronectin (**K**,**L**), showing higher ODs correlated with more intense staining. Quantitative analysis of cell morphology (**M**), regarding cell length (**N**) and aspect ratio (length to width) (**O**) of tenocytes in the presence of HA or in the control medium. (Scale bar: 100 µm).

The staining intensities for collagen I and for fibronectin did not differ between the tenocytes with HA supplementation and the tenocytes in the control medium at day 14 of culture (Figure 7). This finding was true for calculating the OD per cell as well as for the OD per 100,000 cells/mm^2^. Immunofluorescence staining confirmed these findings qualitatively. Cells showed a similar heterogeneous cell morphology, concerning cell length and aspect ratio in both culture conditions.

## 3. Discussion

The formation of fibrous adhesions is a major problem during tendon healing; this ultimately can lead to work disability, pain and general discomfort [1,23]. Adhesions are caused by the laceration of the basement membrane on the tendon surface, where cells migrate out in case of injury. Such cells finally deposit extracellular matrix (ECM) components—together with fibrin—and end up in adhesion formation [24,25].

There are different surgical and therapeutic strategies for addressing adhesions. In clinics, early active motion, thermal therapy, electrical therapy and physiotherapy are used to reduce adhesions [26]. Surgical approaches include physical barriers around conventionally sutured tendons [27]. Further policies are pharmacologically oriented [28], such as using biolubricants in order to enhance the gliding capacity [29]. Among natural lubricants, hyaluronic acid (HA) is highly promising [30,31] because it occurs as the main lubricating agent in the natural synovial fluid [32].

In that sense, electrospun tubes with HA fabricated to address deep flexor tendon ruptures in a sheep model acted successfully to reduce adhesions [33]. Moreover, a cell–hydrogel–fiber composite, which mimics the synovial sheath and promotes endogenous hyaluronic acid formation, has been reported to be promising [34]. Both studies were based on the intrinsic lubricating property of HA. It has to be emphasized, however, that the molecular weight of HA is crucial [12]; HMW HA is reported to be anti-angiogenic, and it downregulates inflammatory responses, while LMW HA shows the opposite effect.

We previously developed anti-adhesion DegraPol^®^ polymer tubes that were tested in a rabbit Achilles full transection model and that helped reduce the adhesion extent by −20% [19,21]. As we intend to further optimize the observed anti-adhesion effect by the incorporation of HMW HA in a second layer adjacent to DegraPol^®^, it is of great interest to know what impacts such HA would have on the tenocytes once the tubular implant degrades over time and the HA is released. Tenocytes are not expected to adhere to the HA layer of the implant, but the cells would interact with dissolved HA molecules released into the peritendinous space.

The major findings were that dissolved HA did not affect rabbit Achilles tenocyte proliferation within 14 days and slightly decreased the gene expression of ECM markers. At the protein level, collagen I and fibronectin expression remained unaffected (Figure 7). Moreover, the gene expression of typical tendon and remodeling markers was approximately unaffected. As for the series of pro-inflammatory marker genes, only *IL-6* was markedly enhanced after stimulation with HA, but was downregulated in the inflammatory environment after LPS induction and HA incubation. These findings can be judged as positive regarding the intention to use HMW HA as a construction material for a barrier tube and lubricating agent to reduce adhesions, as the regeneration of the injured tendon would not be affected by HA.

A closer look at the impact of HA on the gene expression of collagens *Col 1A1*, *Col 1A2* and *Col 3* (Figure 2A) revealed that the three markers were slightly but significantly downregulated after 14 days. Limited matrix formation would serve our final purpose—to reduce adhesion formation—by keeping the ECM production in check. After laceration, adhesive fibrin clots interact with newly formed ECM proteins, and the entangled collagens, fibronectin and fibrin together form an adhesive mixture [7,35]. Thus, the downregulation of ECM gene expression achieved the final aim of anti-adhesion. Zhao and coworkers have reported similar results [36]. During adhesion inhibition by the application of of a PLLA fibrous membrane around a transsected rat Achilles tendon, gene expression of *Col 1* and *Col 3* was significantly downregulated in the specimen with minimal adhesions [36].

Another aspect is the fact that HMW HA acted a macromolecular crowding (MMC) agent [37,38], occupying 0.14% *w*/*v* at 1.4 mg/mL HA used here. Shendi et al. reported that HMW HA (1.5 MDa) in either 0.05% *w*/*v* or 0.5% *w*/*v* (with Ficoll, a typical MMC agent, as a positive control) enhanced *Col 1, 3* and *4* gene expression at the lower 0.055% *w*/*v* HA concentration, but downregulated them at the higher HA concentration (0.5% *w*/*v*) [39]. Moreover, even when 100 µM ascorbic acid was added in the same experiment [39], which would otherwise boost these ECM proteins (without HA) [40], the authors did not find any significant changes in the matrix deposition of these collagen proteins by fibroblasts, underpinning the MMC effect of HA [39].

The presence of HA seemed to be favorable for not only the downregulation of collagens, but also the reduced gene expression of lysyl oxidase (*LOX*) (Figure 2A). LOX binds to several ECM proteins and provokes crosslinking [41,42]. Saifi et al. have reported that LOX inhibition leads to reduced collagen deposition in renal fibrosis [43]. Although renal fibrosis and tendon adhesion are not the same, they share the same fibrotic activity. Moreover, in vitro studies using human hepatic stellate cells showed that the knockdown of LOX suppressed collagen 1 [44], probably via TGF-β/Smad signaling [42]. Hence, the argument of lowered *LOX* expression supporting anti-adhesion is corroborated by such findings as well.

Further suppressive trends in matrix formation were reflected in the downregulation of the genes encoding aggrecan and decorin, a large and a small proteoglycan, respectively (Figure 2B). The typical cartilage proteoglycan aggrecan [45] is also important for tendons because it provides tendon tissue with a high resistance to compressive loads [46]. Interestingly, however, an overexpression of aggrecan is occasionally associated with tendinopathy [47]. Aggrecan tightly interacts with HA to form stable complexes in the ECM. Regarding the equilibrium of this association between aggrecan and HA, it can be speculated that cells exposed to supraphysiological HA concentrations downregulate *aggrecan*, because less extracellular aggrecan is needed to reach a physiological association equilibrium state.

Decorin and biglycan are members of the small leucine-rich proteoglycan family, which are important for collagen fibrillogenesis and matrix assembly [46,48]. While the gene expression of *decorin* was downregulated in the presence of HA, *biglycan* experienced a slight upregulation on day 7 only (Figure 2B). The anti-adhesive properties of decorin and biglycan have been reported for human airway smooth muscle cells (HASMCs) [49]. In contrast to the collagen 1 substrate, where HASMCs exhibited prominent F actin filaments and high focal adhesion kinase (FAK) protein levels, the same cells seeded on decorin or biglycan did not have such filaments and had lower FAK levels—a clear deviation from normal cell adhesion. Hence, the slightly increased *biglycan* gene expression supported anti-adhesion, while the lowered *decorin* gene expression went along with the downregulated matrix production observed for *Col 1*, *Col 3* and *aggrecan*.

The gene expression of typical tendon marker genes was further assessed (Figure 3). Tenascin C (TNC) is a mechanosensitive protein that is prominently expressed in tendons and that binds to several components of the ECM [50], whereas Mohawk is a highly mechanosensing transcription factor important for tendon differentiation during development [51,52]. We found a small but significant downregulation of *TNC* and *MKX* expression on day 3 induced by HA (0.7-fold). Although HMW HA in the culture medium could have impacted the microviscosity, and thus the stiffness, of the microenvironment [53]—also by forming some typical pericellular coatings on the tenocytes [12]—this mechanical impact was only slightly reflected in the *TNC* and *MKX* gene expression and only on day 3, after which the cells seemed to have adapted to the HA.

In contrast to *TNC* and *MKX*, the gene expression of tenomodulin steadily decreased over time, with lower *TNMD* expression on day 14 (0.6-fold) compared to previous time points. Tenomodulin is known to impact tenocyte proliferation [54] and to regulate collagen fibrillogenesis in tendons [55]. The lowest *TNMD* expression in the presence of HA was found on day 14 and paralleled the observed downregulation of *Col 1A1*, *1A2* and *3* (Figure 2A), with slight, but steady and significant, downregulation to a final 0.5-fold expression level. Again, the suppressed matrix production, including reduced collagen fibrillogenesis, supported the anti-adhesion strategy followed here.

The pro-fibrotic marker *α-SMA* was not influenced by HA to a great extent, although the inter-rabbit variability was not negligible (Supplementary Materials, Figure S1). As with *TNC* and *MKX* gene expression, a slight but significant downregulation was measured on day 3, while on day 7, a similarly small and significant upregulation was found—with no further changes on day 14 (Figure 3). Although minimum adhesion formation during Achilles tendon healing in rats has been associated with a downregulation of α-SMA protein expression around the repaired tendon [36], we did not find a prominent downregulation in the presence of HA. In addition, the gene expression of the proliferation marker *ki67* was downregulated on day 3 and remained unaffected thereafter (Figure 4), supporting our aim of utilizing the anti-adhesion properties of HA. Cell proliferation leads to more matrix production, so restricted proliferation should go along with less matrix formation.

As for the remodeling markers, the gene expression of *MMP-2* and *MMP-9*, two prominent gelatinases responsible for basement membrane degradation, was assessed (Figure 4) [56]. While MMP-2 was significantly upregulated on days 7 and 14 (1.4-fold), indicating an increase in matrix turnover, MMP-9 experienced a downregulation on day 3 (0.8-fold), but stayed unaffected compared with the control later on. Atta et al. have shown that increased cleavage of collagen fragments through the overexpression of MMP-9 reduces peritoneal adhesions [57]. Hence, an increase in *MMP-2* and a brief reduction in *MMP-9* that stays otherwise constant overall helps our anti-adhesion strategy through HA. As for the tissue inhibitor of matrix metalloprotease-1 (*Timp1*), we determined a significant downregulation on day 3 (0.8-fold), with no further changes later. This reduction might improve anti-adhesion, as Hassanabad and coworkers have reported adhesion formation through myofibroblasts that overexpressed *collagens 1* and *3*, but also *Timp1* [58].

Furthermore, HA had a prominent impact on pro-inflammatory and pro-resolving *IL-6* gene expression (nine-fold) when artificial stimulation was performed without the situation of tendon injury (Figure 5). In contrast, in the inflammatory environment provoked in vitro by LPS stimulation, treatment with HA resulted in the slight downregulation of *IL-6* (from nine-fold to eight-fold) (Figure 6), which allowed conclusions to be drawn about the response in vivo. IL-6 has been reported to enhance collagen and also fibrinogen, the precursor of fibrin as a main component of fibrous adhesions, in dermal fibroblasts [59]. Moreover, IL-6 drives intraperitoneal fibrosis and supports myofibroblast differentiation, accelerating adhesion formation [60]. In contrast to our findings, Stellavato et al. reported the downregulation of *IL-6* in synoviocytes exposed to HMW HA for 2 days. It has not been reported, however, for longer periods, where *IL-6* dynamics may change.

Although IL-6 is an important pro-inflammatory cytokine during acute initial inflammation, it nevertheless plays roles in resolving inflammation [61,62]. With respect to such pro-resolving characteristics and even some reported anti-inflammatory functions of IL-6 [63], the inflammatory response towards our implant material containing HMW HA needs to be further investigated in an appropriate in vivo environment. In contrast to *IL-6*, however, *TNF-α* and *PAR-2* did not show serious inflammatory side-effects, because they were only slightly impacted (Figure 5). Although TNF-α belongs to the pro-inflammatory cytokines and is expressed more prominently by adhesion fibroblasts compared to normal fibroblasts, it has nevertheless been reported to contribute to the production of IL-10 (anti-inflammatory) and to upregulate a pro-resolving master receptor that transduces annexin A1 and lipoxin A4 [64,65,66].

The gene expression of pro-resolving *ALOX15* (15-lipooxygenase) was slightly downregulated on day 3 under HA exposure, and at later time points there was no effect by HA (Figure 5). Similarly, M2 macrophages have been reported to highly express *ALOX15*, but a downregulation of *ALOX15* was observed under LMW HA treatment [67].

In a pro-resolving milieu mimicked by Lipoxin A4, the tenocytes experienced a small but significant *ALOX15* downregulation (Figure 6D), which was reversed to a significant increase when HMW HA was also present (Figure 6D) It has been reported that, under Lipoxin B4, ALOX15 protein expression was increased in healthy as well as diseased tendon cells [68]. Compared to this report, our experiments were rather short (6 h) and we used Lipoxin A4 rather than B4, which might have influenced the gene expression in tenocytes differently [69]. Nevertheless, HA mitigated this unexpected effect of Lipoxin A4. In line with this positive effect induced by HA, *ALOX15* was also significantly increased under LPS with additional HA.

As for the pro-inflammatory markers, the tenocytes experienced a prominent upregulation of *IL-6* and *PAR-2* under pro-inflammatory LPS conditions as expected, while *TNF-α* was not affected (Figure 6A–C). Obviously, *TNF-α* regulation was less sensitive to the pro-inflammatory environment mimicked by LPS compared with *IL-6* and *PAR-2*. Regarding the HA-only exposition on day 3 (Figure 5), *IL-6* was also upregulated, while *TNF-α* was not affected. However, the impact on *PAR-2* was the opposite; HA stimulation led to a slight *PAR-2* downregulation on day 3 (0.8-fold) (Figure 5), while under LPS it was significantly upregulated (1.9-fold) (Figure 6C), indicating that HMW HA did not evoke a general pro-inflammatory response in the tenocytes (as LPS did), but selectively affected *IL-6*.

Some limitations of the study have to be mentioned. First of all, we used only one type of HMW HA, albeit a commercially available one with a wide range of sugar chain lengths. HMW HAs with even longer chain lengths would be worthwhile to compare. Second, LMW HA would be interesting in a general sense, although not for our future objective of using HA for implant electrospinning. Moreover, we determined the dynamics of tenocyte gene expression at 3, 7 and 14 days and at only one HA concentration; shorter and longer cell culture experiments with different concentrations would be interesting, the latter also with respect to ECM formation in the future. Finally, we chose only one cell type (rabbit tenocytes); however, synoviocytes and mesenchymal stem cells, as well as fibroblasts and myofibroblasts, would be additional candidates—which could also be obtained from species other than rabbits—that play pivotal roles during tendon healing and that might be affected by HA.

## 4. Conclusions

In summary, we demonstrated that rabbit Achilles tenocytes exposed to 1.4 mg/mL of HMW HA experienced a general downregulation of matrix markers, crosslinking *LOX*, typical tendon markers, *α-SMA* and *ki67* (the latter only on day 3). On a protein level, collagen I and fibronectin remained unaffected. These findings suggest that HMW HA is a good choice to be used as an anti-adhesive material to fabricate bi-layered tubes for tendon repair, acting as a physical barrier with a non-adhesive surface on the outer layer. Upon the degradation and therefore the release of HMW HA, such tubes will not provoke a fibrotic reaction, but rather prevent adhesion formation. A noteworthy result was that the pro-inflammatory and pro-resolving cytokine *IL-6* was enhanced, while other pro-inflammatory markers remained unaffected. HA stimulation in the presence of LPS mitigated pro-inflammatory and enhanced pro-resolving marker genes. These findings pave the way for a biodegradable anti-adhesion implant material based on HMW HA in the future.

## 5. Materials and Methods

### 5.1. Isolation of Rabbit Achilles Tenocytes and Cell Culture

Rabbit tenocytes were isolated from ATs of three New Zealand White rabbits using the cell migration method (approval by the veterinary office of Canton Zurich, reference number 255/15). Briefly, tendons were extracted from the animals and washed with PBS (Sigma-Aldrich, # D8537, Merck, Buchs, Switzerland) supplemented with 200 µg/mL gentamicin (Biowest, # L0011, Nuaillé, France) and 2.5 µg/mL amphotericin B (Pan Biotech, # P06-01100, Aidenbach, Germany). Tendons were cleaned from the surrounding tissue and the central part of the tendons was cut into very small pieces (<2 mm) and washed three times in PBS buffer. Afterwards, multiple tissue pieces were placed into a tissue culture plate (PrimariaTM, Corning, New York, NY, USA) and a drop of cell culture medium was added onto each tissue piece (Ham’s F12 (Biowest, # L0135-500, Nuaillé, France); 10% FBS (Biowest, # S1830-500, Nuaillé, France), 100 U/mL penicillin, 100 µg/mL streptomycin (ThermoFisher scientific # 15140122, Basel, Switzerland) and 1% GlutaMAX™ (ThermoFisher # 35050038, Basel, Switzerland)). Tissues were allowed to attach onto the cell culture plates for 2 h at 37 °C and 5% CO_2_ before adding 10 mL of cell culture media into each plate.

The plates with the tissues were not moved for the first 5 days, to decrease tissue detachment upon plate movement and to allow cells to start migrating out from the tissues. The first medium change was performed after 5 days, and subsequently, the culture medium was changed every third day. After approximately 2 weeks, tissue pieces were removed from the plates, and cells were allowed to proliferate for 1 week more before cryopreservation. Cryopreserved rabbit tenocytes were thawed, resuspended in culture medium and cultured at 37 °C and 5% CO_2_, with the media being changed every second day. Tenocytes between passages 2 and 4 (P2–4) were used for all experiments. The same procedure has been reported before [40].

### 5.2. Cell Proliferation

Cell proliferation was determined on day 3, 5, 7 and 14 by alamarBlue™ cell viability assay (Invitrogen, # DAL1100, ThermoFisher Scientific, Basel, Switzerland). An amount of 350 cells/well in technical triplicates was transferred into a 96-well plate (TPP, # 92096, Trasadingen, Switzerland) in 100 µL of culture medium with or without 1.4 mg/mL of HA supplementation (Lifecore Biomedical, # HA 15M, Chaska, MN, USA), and was cultured at 37 °C with 5% CO_2._ A culture medium containing HA was prepared and exchanged daily, and the medium without HA was exchanged every second day. Empty wells were filled with PBS to prevent dehydration. The alamarBlue™ solution was diluted to 1:10 in cell culture medium and incubated for 4 h on the cells before the excitation wavelength of 530 nm and emission wavelength of 590 nm were measured using a Cytation 5 imaging reader (BioTEk, Agilent Technologies AG, Basel, Switzerland). Standard curves were made to correlate fluorescence intensities to cell number. A calculation of doubling times (DT) was performed according to doubling time = number of days × 24 h/(log factor of population increase/log2).

### 5.3. Real-Time PCR

In order to determine the effect of HA supplementation on the gene expression of tenocytes in vitro over time, rabbit tenocytes were isolated and cultured as described under Section 5.1. and seeded into 6-well plates (Sigma-Aldrich, # SIAL0516, Merck, Buchs, Switzerland; growth area per well: 9.6 cm^2^) with a density of 2 × 10^5^ cells/well in 2 mL of culture medium. Cells were allowed to attach overnight before the culture medium was exchanged with a medium either containing 1.4 mg/mL of HA or without any supplementation. The desired HA concentration was freshly added to the culture medium daily for a total of 2 weeks. Samples were collected after 3, 7 and 14 days. Rabbit tenocytes from three different animals were used (n = 3) and cell culture experiments and qPCRs were performed in triplicate for each rabbit.

In order to determine the effect of HA on immune responses, lipopolysaccharides (LPS) (Sigma-Aldrich, # L4391, Merck, Buchs, Switzerland) were used to enhance immune reactions and Lipoxin A4 (Sigma-Aldrich, # 437725, Merck, Buchs, Switzerland) was added to the culture medium in order to resolve inflammatory reactions and mimic resolution. Cells were seeded into 6-well plates and allowed to attach as described in the text section above. Tenocytes were treated with Lipoxin A4 (10 ng/mL) for 6 h, with LPS (1 µg/mL) for 4 h or with a combination of both metabolites (4 h LPS followed by Lipoxin A4 for 4 h). Rabbit tenocytes from three different animals were used (n = 3) and cell culture experiments and qPCRs were performed in triplicate.

At the respective time point, total RNA was isolated using the RNeasy Plus Mini Kit with RNase-free DNase treatment (Qiagen, # 74136, Hilden, Germany), following the manufacturer’s protocol. The amount and purity of RNA was measured with Nanodrop One (ThermoFisher, Basel, Switzerland). For reverse transcription (RT), 500 ng RNA was investigated in a reaction volume of 20 µL (SuperScript III Reverse Transcriptase, # 18080085, ThermoFisher, Basel, Switzerland; Oligo(dT)12-18 Primer, # 18418012, ThermoFisher, Basel, Switzerland; RNase Inhibitor, # N8080119, Applied Biosystem, ThermoFisher, Basel, Switzerland; dNTP, # 18427013, Invitrogen, ThermoFisher, Basel, Switzerland) using a compact thermocycler (Masterscycler personal, Eppendorf, Germany). Real-time PCR reactions were performed in technical triplicates with 4 µL of the resulting cDNA using Quant Studio 5 (Applied Biosystems, ThermoFisher, Basel, Switzerland) and Fast SYBR^TM^ Green Master Mix (ThermoFisher, # 4385612, Basel, Switzerland). The samples were heated to 95 °C for 3 min, followed by 40 cycles of 95 °C for 3 s and 60 °C for 20 s. All primers were synthesized by Microsynth, Balgach, Switzerland (Supplementary Materials, Table S1). A relative expression analysis was performed using the comparative 2^−ΔΔCT^ method with *18S* as a reference gene, which was stable over the two conditions analyzed. Results are presented as fold changes normalized to the control, i.e., compared to samples cultivated without HA (set to 1).

### 5.4. Pro-Inflammatory and Resolution Markers

In order to test the effect of HA supplementation on tenocytes regarding collagen I and fibronectin production in vitro, cells were seeded into 2-well culture slides (BD Falcon, # 354102, Schaffhausen, Switzerland) at 1 × 10^5^ cells/mL (2 mL per well, growth area per well: 4 cm^2^) in a culture medium as described in Section 5.1. Cells were allowed to attach overnight before the culture medium was exchanged to medium with HA (1.4 mg/mL) or without any supplementation. The desired HA concentration was added to the medium daily for a total of 14 days, and the culture medium was exchanged every second day.

For immunocytochemical experiments, samples for collagen I and fibronectin were cultured separately and staining was carried out with standardized protocols, using Autostainer Link48 (DAKO, Basel, Switzerland). Shortly, for DAB staining, slides were fixed in Delaunay fixation solution (Morphisto, # 16001, Biosystems, Muttenz, Switzerland) for 1 min and treated with 3% H_2_O_2_ for 10 min. After washing with washing buffer (DAKO, # K800721, Baar, Switzerland), samples were blocked with serum for 30 min and primary antibody was added for 1 h. After the next washing step, DAB (DAKO, # K3468, Baar, Switzerland) was added for 10 min followed by Hematoxylin treatment (DAKO, # K8008, Baar, Switzerland) for 10 min. Samples were rinsed with washing buffer before they were transferred into water and dehydrated with ethanol. For collagen I staining, a biotin goat polyclonal antibody to collagen I was used (abcam, # ab24821, Cambridge, UK, 1:100 dilution) and blocking was carried out with horse serum (Vector, # S21000, AdipoGen, Liestal, Switzerland). For fibronectin, a mouse monoclonal antibody to fibronectin was used (Sigma-Aldrich, # F0791, Merck, Buchs, Switzerland; 1:200 dilution) and samples were blocked with goat serum (Vector, # S-1000, AdipoGen, Liestal, Switzerland). Samples from three isolations in technical triplicates for each condition were imaged with confocal microscopy (LEICA DM6000B, Heerbrugg, Switzerland) equipped with a digital camera. In each histological section, three fields of view (FOV) at 25× magnification were analyzed quantitatively according to the mean grey value using the Colour Deconvolution function in ImageJ 1.52a. For cell morphology, 200× magnification was used and the length and width of cells lying on the diagonal in three FOVs for each section were measured.

For immunofluorescence staining, cells were fixed in Delaunay fixation for 1 min and then washed with washing buffer before samples were blocked with donkey serum (abcam, # ab7475, Cambridge, UK) for 30 min. After washing, primary antibodies were added for 1 h and then slides were washed again before adding a secondary fluorescent antibody. After a next washing step, slides were mounted with a mounting medium containing DAPI (Vector, # H-1500, AdipoGen, Liestal, Switzerland). The same primary antibodies with the same concentration were used as described for DAB staining in the preceding text section. For collagen I, a donkey anti-goat Alexa-488 secondary antibody (ThermoFisher Scientific, # A32814, Basel, Switzerland; 1:1000 dilution, 20 min incubation time) was used. The fluorescence staining for fibronectin was carried out with a donkey anti-mouse Alexa-488 secondary antibody (ThermoFisher Scientific, # A21202, Basel, Switzerland; 1:1000 dilution, 30 min incubation time). Tenocytes from two isolations in technical duplicates were imaged for qualitative analysis at 200× magnification using a confocal microscope.

### 5.5. Statistical Analysis

Data were analyzed with Graph Pad Prism 9 software. An unpaired *t* test was performed for differences between two groups, for example with or without HA. *p* values < 0.05 were considered significant and indicated by *; *p* < 0.01 (**) and *p* < 0.001 (***). Values were expressed as means ± standard deviations.

## Data Availability

Not applicable.

## Supplementary Materials

The following supporting information can be downloaded at: https://www.mdpi.com/article/10.3390/ijms23147926/s1.
