# Supplementary material for: Impact of High-Molecular-Weight Hyaluronic Acid on Gene Expression in Rabbit Achilles Tenocytes In Vitro
Source: Int J Mol Sci. 2022 Jul 18;23(14):7926. doi: 10.3390/ijms23147926 (PMC9320370; doi:10.3390/ijms23147926)
Supplement: Supplementary file 1 [file ijms-23-07926-s001.zip › ijms-1822050-supplementary.pdf]

Supporting Information

Impact of High-Molecular-Weight Hyaluronic Acid on Gene Expression in Rabbit Achilles Tenocytes In Vitro

Iris Miescher [1], Petra Wolint [1], Christine Opelz [1], Jess G. Snedeker [2], Pietro Giovanoli [1], Maurizio Calcagni [1] and Johanna Buschmann [1,*]

[1] Division of Plastic Surgery and Hand Surgery, University Hospital Zurich, Sternwartstrasse 14, 8091 Zurich, Switzerland; iris.miescher@usz.ch (I.M.); petra.wolint@usz.ch (P.W.); christine.opelz@web.de (C.O.); pietro.giovanoli@usz.ch (P.G.); maurizio.calcagni@usz.ch (M.C.)
[2] Orthopaedic Biomechanics, University Clinic Balgrist, Forchstrasse 340, 8008 Zurich, Switzerland; jess.snedeker@hest.ethz.ch
* Correspondence: johanna.buschmann@usz.ch; Tel.: +41-44-255-98-95

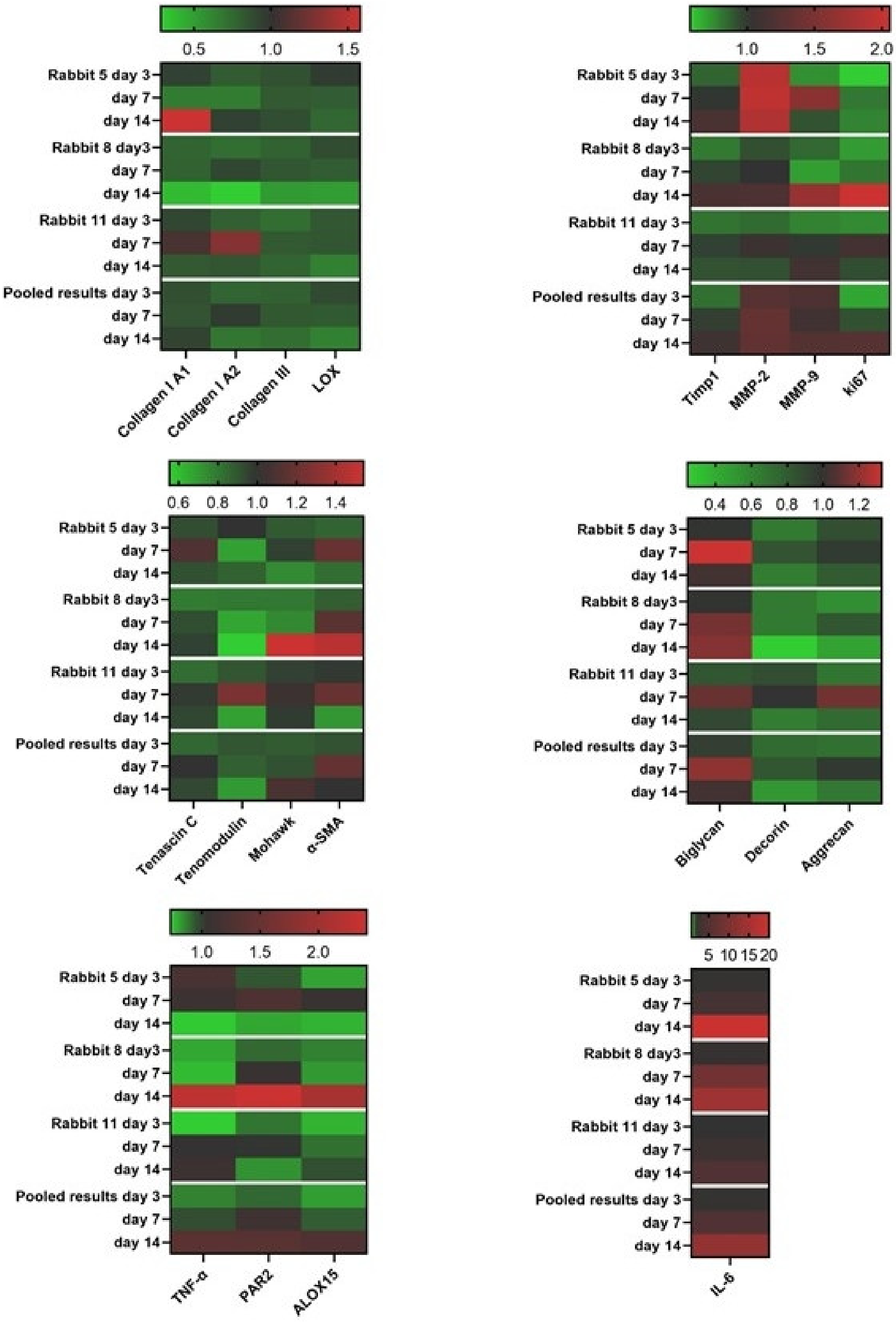

Figure S1. Heat Maps of qPCR results from tenocytes isolated from Achilles tendon of three different rabbits. Fields in green show downregulation of gene expression level, fields in red overexpression and black rectangles show no difference compared to cDNA isolated from tenocytes cultured in control medium without HA.

Table S1. Gene abbreviations, NCBI reference sequences of mRNAs (used as template for primer design), amplicon size and primer sequences used for Real-Time PCR Analysis.

Gene	mRNA ID (NCBI Reference Sequence)	Amplicon Size (bp)	Forward Primer Sequence (3′→ 5′)	Reverse Primer Sequence (5′→ 3′)
**18s rRNA**	NR_033238.1	176	GGAACTGAGGCCATGATTAAG	CGGAACTACGACGGTATCTG
**Col1A1**	XM_008271783.1	271	CTGGTGAATCTGGACGTGAG	TGTCTCACCCTTGTCACCAC
**Col1A2**	NM_001195668.1	170	CCAATCACGCCTCTCAGAAC	GCAGCCATCGACAAGAACAG
**Col3A1**	XM_002712333.2	259	GCATTGCTTACATGGATCAGG	CCAACGTCCGCACCAAATTC
**LOX**	XM_008254949.1	176	TCCAGCCCGGCAACTACATT	TATCCGTTGGGAGTTGGGGC
**TNC**	FJ480400.1	219	GTCACTCATCACAGCTCTGG	CTGAGTGTGTATTCCGTGGC
**TNMD**	NM_001109818.1	239	GCAGTTTCCGAGTTACAAGAC	CGACGGCAGTAAATACAACAG
**DCN**	NM_001082330.1	255	GTGGACAATGGTTCTCTGGC	AAGGTGGATGGCTGGATCTC
**ACAN**	L38480.1	113	GCTACGGAGACAAGGATGAG	GTAAAAGACCTCACCCTCCAT
**BGN**	NM_001195691.1	199	GGCCTGAAGCTCAACTACCT	GGCTCCCGTTCTCAATCATC
**Mohawk**	NC_013684.1		AACGTCGAGCAGTCTCTGAG	CACGCACTCTGGTACAGTTG
**α-SMA**	NM_001101682.2	224	CGTGACTACTGCTGAACGTG	GGATGCCAGCAGATTCCATC
**MKI67**	XM_008251084.2	283	CACATCCAGCAGTGAAACGG	GTGTTAGCAGTACCTGAAGTC
**TIMP1**	NM_001082232.2	236	CTACCTTGTACCAGCGTTATG	GAAGCTCAGACTGTTCCAGG
**MMP2**	D63579.1	220	GAAGATCGACGCTGTGTACG	GTATCTCCAGAACTTGTCTCC
**MMP9**	D26514.1	160	GATACAGCCTGTTCCTCGTG	GGACCATATAGATGCTGGATG
**IL-6**	AF169176	153	GAAAACACCAGGGTCAGCAT	CAGCCACTGGTTTTTCTGCT
**TNF-α**	M12845	138	CTCCTACCCGAACAAGGTCA	CGGTCACCCTTCTCCAACT
**PAR2=F2rl1**	NW_003159481.1		TTCCCGGCCTTCCTCACGG	CTTCTGGGAGCTGAGC
**ALOX15**	NM_001082282.1	141	CGACTTCGCCCTGCTGGATA	GCTGGATGACCATAGGCATGA
**CD206**	XM_002717356.3	198	TGTCCAGAGGATTGGGGCAC	TTCATGGTAGCTCCCGCCAG

Abbrevations: **TNC** = tenascin C, **TNMD** = tenomodulin, **DCN** = decorin, **ACAN** = aggrecan,

BGN = biglycan, **MKI67** = Ki67
